# 3-D Space Visualization System Using Ultrasonic Sensors as an Assistive Device for the Blind

**DOI:** 10.1109/JTEHM.2020.2978842

**Published:** 2020-03-12

**Authors:** Jung-Hun Kim, Ji-Eun Park, Jong-Min Lee

**Affiliations:** School of Electronics EngineeringKyungpook National University 34986 Daegu41566South Korea

**Keywords:** Auditory signal, visual disturbance, visual reconstruction, vision system

## Abstract

This study proposes a new assistive device for the blind that uses more than one-dimensional data to draw objects. The study aims to convert three-dimensional (3-D) spatial information into sound information using 6-axis and ultrasonic sensors, and to draw a 3-D depiction of the space ahead for the user. Fourteen participants were involved in testing, wherein 4 were visually impaired. Moreover, the male to female ratio was 7:3, with the average age of participants at 28.8 years. An initial sound recognition experiment was designed to assess the device’s accuracy through participant use. Recognition rates were 70% for normal participants and 88% for the blind participants. Additional experiments expanded the environmental conditions by requiring participants to discern the distances of 10 objects, positioned at both high and low locations. Two different scenarios were employed: stationary and walking scenarios. The stationary distance measurement participants scored an average of 96 points, while the walking participants averaged 81 points. Under the given conditions, this study found that its assistive device for the visually impaired can draw a 3-D space with 88.5% accuracy. This probability promises a basic level of utility that can assist those with visual impairment in controlled environments, such as hospitals and homes.

## Graphical abstract

This study proposes a new assistive device for blind people that converts three-dimensional (3-D) spatial information into sound information using 6-axis and ultrasonic sensors, and then draws a 3-D depiction of the space ahead for the user. Human testing found that this assistive device for the visually impaired can draw a 3-D space with 88.5% accuracy, a basic level of utility that can assist those with visual impairment in controlled environments, such as hospitals and homes.

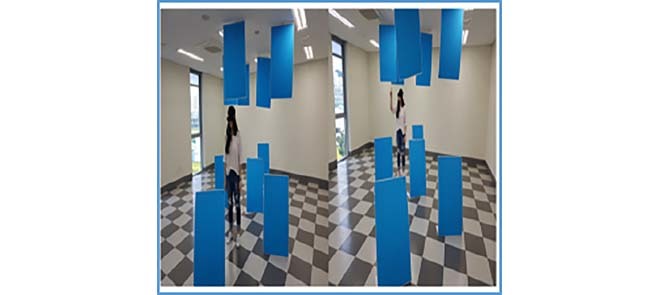

## I. Introduction

Development efforts for assistive devices for people with disabilities (PWD) have become more active than ever [1]. Various ancillary equipment enabling PWDs to engage in physical and social activity, and overcome daily obstacles are in development. The propagation of these devices has had positive effects on the lives of PWDs who need assistive devices [2]. The recent technological advances have allowed the research and development of assistive devices to progress, both domestically and internationally, steadily [3]–[5].

This study offers one potential alternative by proposing a method to compensate for any visual impairment. As an essential bodily function, one’s vision is responsible for both spatial and object recognition, also contributing to the individual’s sense of balance. Some studies have tested infrared-based obstacle detection systems; these are developed for indoor use, and they improve user mobility by signaling when obstacles are ahead of the user [6]. Others explored the utility of the Global Positioning System (GPS) in an assistive device, supplemented by ultrasonic sensors, notifying users through signaled vibrations. [7]. Another employs radio-frequency identification (RFID) to provide the location and information of objects [8]. Inspired by Microsoft’s Kinect system, one device attaches to canes and acquires visual three-dimensional (3-D) information to detect front-laying objects through the use of a vibration sensor [9], [10].

However, existing assistive devices are limited to one form of information transmission in their obstacle recognition—making a sound when there is an object in front of it. There has yet to be one that addresses their fundamental problem: their inability to describe a 3-D environment precisely. Thus, in this study, 3 octaves Do, 4 octaves Do, 5 octaves Do, high sound, middle sound, and low sound are all used to draw a 3-D space in front of the user. These sounds are used to determine the distance and location of objects by coming as feedback to the user, adding an element of depth to the sensor’s feedback, and indicating position with corresponding sounds. As no other device uses such feedback to cue spatial information to users, we evaluate a newly patented differentiated assistive device that relays the spatial information with sound-based feedback.

## II. Materials and Methods

### A. Materials

The study was approved by the Institutional Review Board (IRB 2017-0079) of the Kyungpook National University, compliant with regulations. Experiments were conducted using auxiliary devices for the blind as glasses. To ensure participant safety, a security agent was placed in front of the participant, and a nurse was always present in the event of an emergency. Out of the 14 participants, 4 were visually impaired, while the remaining 10 had no abnormalities in hearing and sound recognition. The participants’ gender ratio was 7:3 (male to female), with an average age of 28.8. The participants consented to have their eyes covered for specific parts of the experiment.

### B. Methodology

**FIGURE 1.**
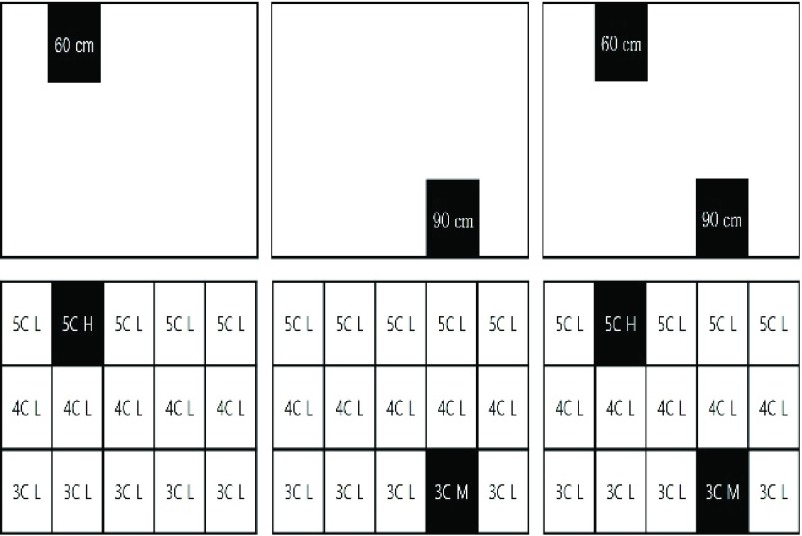
Recognition method of the 3-D space around the object (from left to right: top, 60 cm distance; bottom, 90 cm. * Do refers to Do of the diatonic scale 3C = 3 octaves Do 4C = 4 octaves Do 5C = 5 octaves Do of diatonic H = high sound at 60-cm distance M = middle sound at 90-cm distance L = low sound at 120-cm distance.

Spatial awareness is crucial for walking; for assistive devices with ultrasound, determining objects at a distance from each other is an essential cognitive factor. The maximum range was set to 210 cm to eliminate superimpositions due to the radiation angle of the ultrasonic sensor. To have this system map 210 cm of the room ahead, the ultrasonic wave was subdivided into three levels (i.e., top, middle, and bottom) to precisely locate an object. When the system detects an object across all three levels, each would simultaneously transmit the distance information. Fig. 1 shows how the device recognizes 3-D space:

In a mapped 3-D space, the object is recognized using 3C, 4C, or 5C octaves; a different pitch sounds depending on the distance and position of the object (Fig. 1). The volume of those notes’ ranges from levels 1–10, depending on the distance of the user to the object. At 0 cm, the volume is at level 1; at the 210 cm limit, the volume reaches level 10. For example, if a bottom-lying object is 30 cm away, the system will sound a 2-step volume of 3C octaves; if set at center height and 60 cm away, the system will sound a 3-step volume of 4C octaves; if found at the top height and 90 cm away, the system sounds a 5-step volume of 5C octaves. This system is designed to help users recognize 3-D spaces by adjusting the pitch and volume corresponding to the varying distances of obstacles.

The roll, pitch, and yaw values are outputs of the attitude and heading reference systems (AHRS) 6-axis sensor. Roll indicates horizontal values (X-axis coordinates) while yaw represents the slope values; pitch measures the vertical values used to obtain the Y-axis coordinate of the frontal object. Using the distance value of the ultrasonic sensor and the Y-axis coordinate of the AHRS 6-axis sensor allows the construction of a distance map.

**FIGURE 2.**
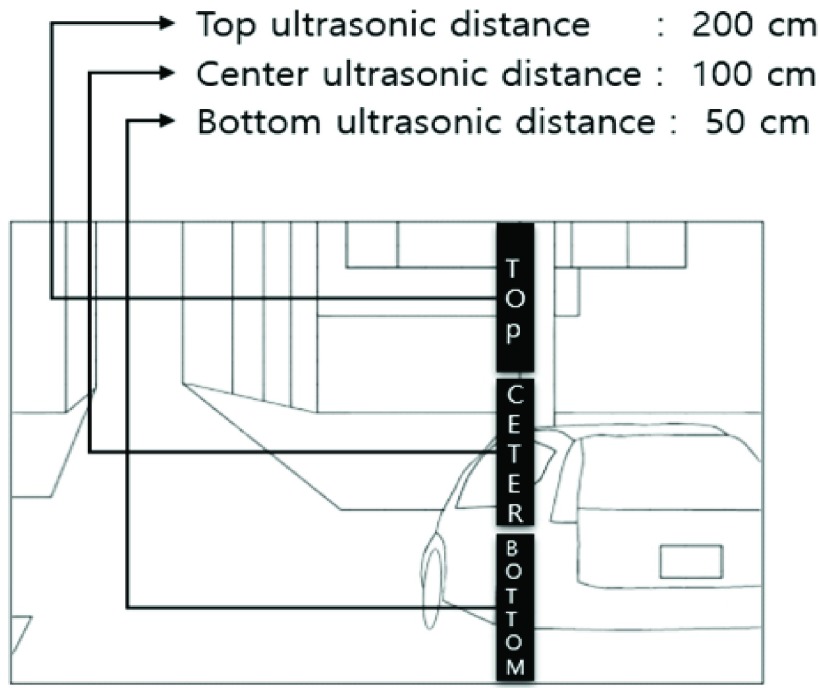
Method of acquisition information in three-dimensional space through three ultrasounds (TOP, CENTER, BOTTOM). TOP: TOP indicates the position of the ultrasonic sensor. The distance of the ultrasonic sensor is 200 cm. 200 cm is the distance of the building. CENTER: CENTER indicates the position of the ultrasonic sensor. The distance of the ultrasonic sensor is 100 cm. 100cm is the distance of the front door of the car. BOTTOM: BOTTOM indicates the position of the ultrasonic sensor. The distance of the ultrasonic sensor is 50 cm. 50cm is the distance of the back of the car.

Fig. 2 shows how the distance information of the 3-D space was obtained using the collected data from the top, middle, and bottom ultrasonic sensors. The collected distance information from the ultrasound has 10 steps as subdivisions: 30, 50, 70, 90, 110, 130, 150, 170, 190, and 210 cm (depending on the distance). The actual detection distance of the ultrasound reaches a maximum of 700 cm. However, if the detection range of the object widens, unnecessary sensory information of various objects not within the immediate proximity of 210 cm (e.g., cars, people, animals, and so on) would register. Therefore, the scope of detection was set to 210 cm.

**FIGURE 3.**
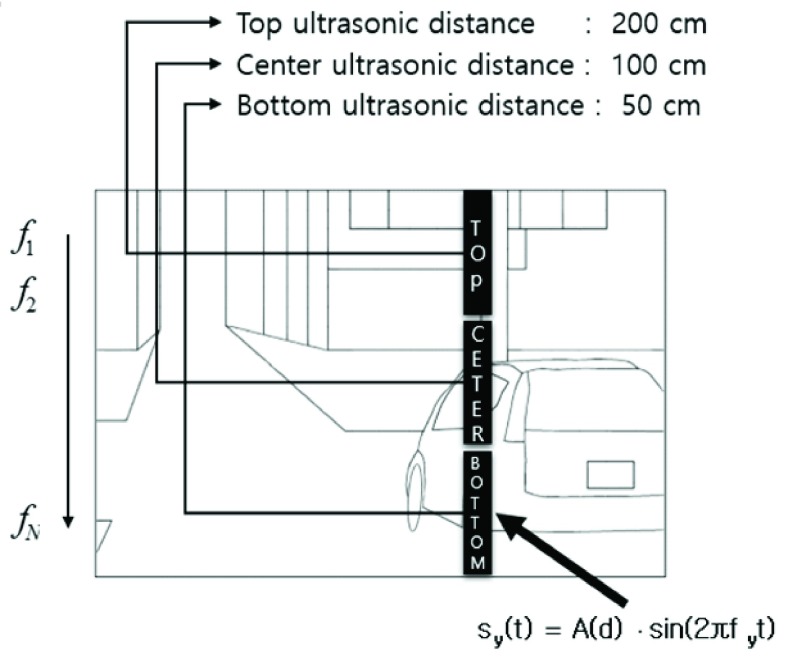
Transformation computation of 3-D information to auditory information. $f$: $f$ represents the frequency and from the high frequency $f$ to the low frequency ($ f_{\text {N}}$) A: Indicates the magnitude of the frequency. At a distance of 200 cm, the frequency is small amplitude. 50cm has a large amplitude-frequency when the distance is close. Sin: A sine wave was used.

As demonstrated above, specific sounds were generated by changing the amplitude $A$ and the frequency $f$ from the fundamental sinusoidal wave:

\begin{align*}&S(t)=A\times \sin (2\pi \textrm {ft)} \\&\textrm {S(t): sound} \\&A:amplitude \\&\textrm {sin(2}\pi \textrm {ft):sine wave}\tag{1}\end{align*}

The distance measured via ultrasound can be expressed by gauging the intensity of the sound:

\begin{align*}&S(d,t)=A(d)\times \sin (2\pi \textrm {ft)} \\&\textrm {S(d,t): sound} \\&A(d): \textrm {distance information value} \\&\textrm {sin(2}\pi \textrm {ft):sine wave}\tag{2}\end{align*}

The frequency according to the height change Y of the object is expressed as follows:

\begin{align*}&S_{S} (t)=\sum \nolimits _{y=1}^{N} {A(d)} \times \sin (2\pi f_{y} t) \\&S_{S} (t)\textrm {: sound} \\&\sum \nolimits _{y=1}^{N} {A(d)}:{\textrm {2-D distance information value}} \\&\sin (2\pi f_{y} t)\textrm {:sine wave with {y-axis} information}\tag{3}\end{align*}

Finally, the 3-D space information is converted into auditory information by changing frequencies using the difference in sound volume according to the signal of the distance and the three levels of the ultrasound.

**FIGURE 4.**
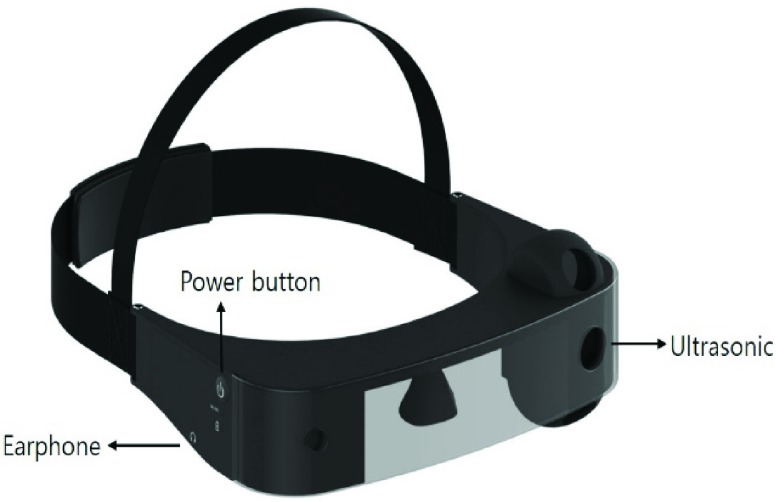
Assistive device model. (It consists of a power button, earphone, and three (TOP, CENTER, BOTTOM) ultrasonic sensors).

Figure 4 illustrates the visor-like device designed for this experiment. Its visor design was done to match conventional eyewear, eliminating bias and mimicking the general user interface. The top, middle, and bottom ultrasounds are located at the left side of the device, while the distance information is collected at the front of the visor.

**FIGURE 5.**
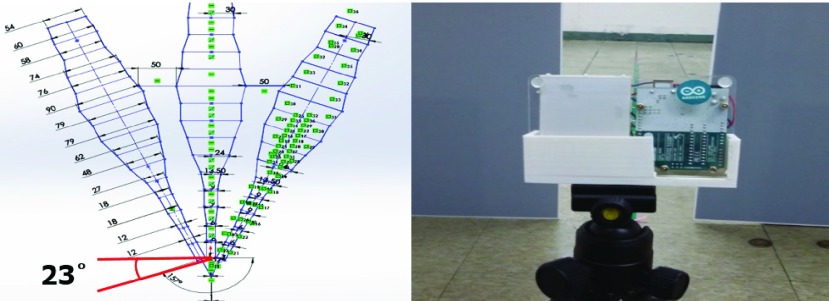
To prevent the interference of ultrasonic waves, ultrasonic profiles were made. To prevent the interference of the ultrasonic wave is manufactured by tilting 23°.

This layout prevents signals from interfering with each other’s transmission and reception. According to the experiment result of Fig. 5, the slope of the TOP, CENTER, and BOTTOM ultrasonic sensor tilted 23°.

**FIGURE 6.**
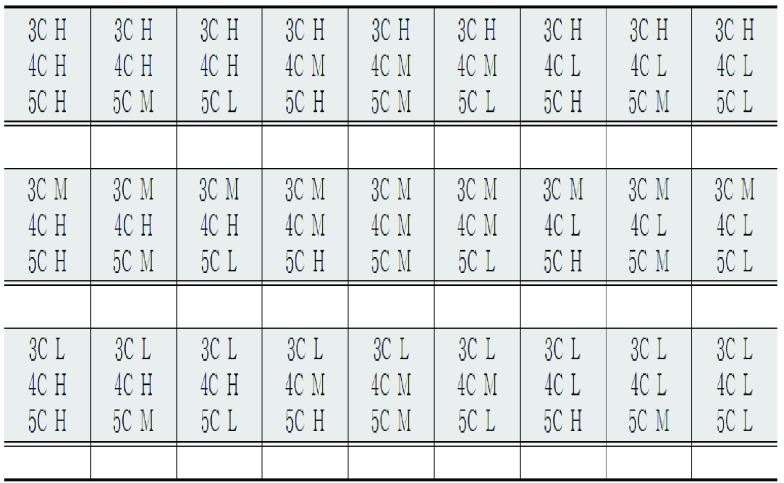
Participants determined whether they could recognize several sounds at the same time. Three octaves (3C, 4C, 5C) were tested with varying volumes. * Do refers to Do of the diatonic scale 3C = 3 octaves Do 4C = 4 octaves Do 5C = 5 octaves Do of diatonic H = high sound at 60-cm distance M = middle sound at 90-cm distance L = low sound at 120-cm distance.

Fig. 6 distinguishes the object position via the assigned octaves and volume. Twenty-seven sound recognition tests were conducted on the 10 participants 90 minutes after the experiment orientation. Section III-A discusses the test results.

**FIGURE 7.**
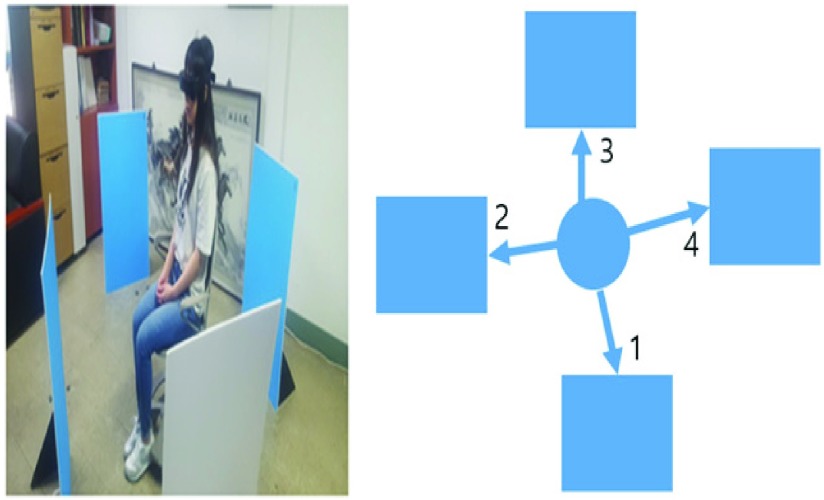
Indoor distance measurement experiment. The experiment was conducted to recognize the distance of each object in the room. Participants identified the objects one to one, from 1 to 4.

**FIGURE 8.**
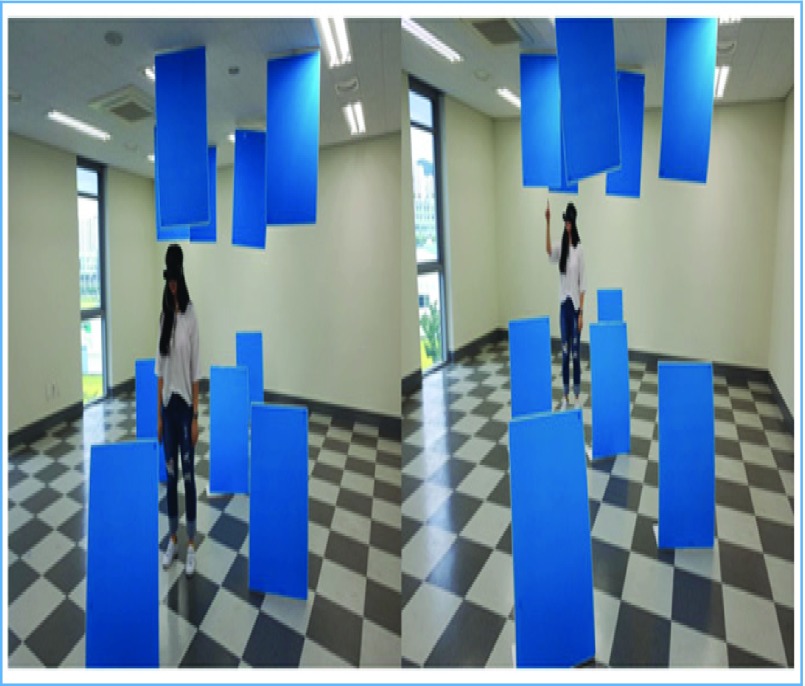
The participant was instructed to move past the obstacles using the assistive device. While moving, the participants had to locate the objects by hand.

While seated, participants were instructed to pinpoint the presented objects through the device and list them from nearest to farthest. After measuring, the distance of the object would be changed. From an initial score of 10 points, 1 point was deducted for every wrong answer. The participants were given 30 minutes to practice after orientation before the experiment proper. The test was subsequently performed 10 times. Section III-B discusses the scores of these trials.

The distance recognition experiment proceeded as follows: first, the participant was oriented on the different sounds the device emitted. Then, the participant equipped the device and moved forward, pinpointing obstacles from the initial location. As the participant drew closer to the object, they pointed at it to demonstrate obstacle recognition. At the end of the course, they removed the blind assistive device with the help of a security guard. Section III-C discusses the test results.

## III. Results

### A. Sound Recognition Experiment

The 10 normal participants averaged 19 out of the 27 (70.37%) objects. On average, when a participant perceives sound, they needed more 180 minutes to distinguish between all 27 sounds and achieve satisfactory results. Based on these results, 3-D space recognition can work with the assigned sounds for position information.

### B. Distance Measurement Experiment in Normal Person

The distance measurement results (Table 1) did not display any significant difference between each other. The high scores imply that the device and its mechanics are intuitive, and the different sounds emitted are easily distinguishable.

**TABLE 1:** Distance Measurement of Objects in 3-D Space Using Sound Volume

Participant	Score	Participant	Score
Participant 1	10	Participant 6	10
Participant 2	9	Participant 7	9
Participant 3	10	Participant 8	10
Participant 4	9	Participant 9	10
Participant 5	9	Participant 10	10
Total		96/100

**TABLE 2:** Experimental Study of 3-D Space Using Sound Volume

Participant	Score	Participant	Score
Participant 1	8	Participant 6	9
Participant 2	8	Participant 7	9
Participant 3	7	Participant 8	8
Participant 4	8	Participant 9	8
Participant 5	8	Participant 10	8
Total		81/100	

### C. Experimental Method for Interior Obstacles EN Route

The results of the experiment using upper and lower obstacles have lower results compared to the other experiments. However, despite that gap with other experiments, the discrimination of distance and location in the 3-D space has approximately 81 % recognition rate

## IV. Discussion

While previous studies used auxiliary devices with ultrasonic, camera, or infrared sensors to analyze surrounding obstacles, the assistive devices designed here use converted data to help the blind navigate past peripheral obstacles [11]–[15]. Although there are viable data among the different sensors available, ultrasound proves most useful in discriminating distances. Certain external circumstances (i.e., the camera sensor commits errors depending on the shape/pattern, infrared rays are not suitable for outdoor use) limit cameras and infrareds; thus, ultrasonic sensors are the recommended tool for developing these assistive devices.

However, ultrasound can only discriminate distance, and ultrasonic sensors cannot recognize such overall space. The device developed for this study creates a two-dimensional plane coordinate using a 6-axis sensor and draws that missing 3-D space, taking a step above existing models.

With normal participants attaining recognition rates of 70% respectively and the distance measurement experiment scoring 96 points and the combined indoor walking test scoring 81 points implies that 81% of the given 3-D space is recognizable through the device. However, as the distance is limited to a 210-cm range, the obtained information of 3-D space is restricted in turn. Longer distances might be measured using a higher frequency ultrasonic sensor with a smaller radiation angle. Nevertheless, the proposed assistive device employing a 6-axis sensor, a high-frequency ultrasonic sensor, and GPS to locate positions is expected to perform more accurately compared to other assistive devices for the blind.

Visually impaired people have more sensitive hearing than individuals without any impairment [16]. By using these echolocation functions, the device makes it possible for persons with this particular disability to identify better their surrounding environment and the obstacles that lay before them. Especially useful is the distance discrimination the device employs, allowing users to distinguish the location and distance of objects ahead. If a person with any visual impairment is equipped with this device, based on our findings, they will be more likely to recognize and adapt to any front-laying obstacles; this may prove especially useful in controlled environments such as hospitals and homes with its portability and relatively compact functionality.

Future studies might compare the effectivity of such a device versus the more traditional equipment to assist persons with any visual impairment. Also, these devices might be adapted to more real-life simulations by adding the recognition of moving obstacles as objects of study, or a combination of object behaviors in their testing conditions.

## V. Conclusion

This new device addresses issues previous iterations of similar assistive devices faced by utilizing a different data acquisition method. The 6-axis and ultrasonic sensors gather data that, when used together, allows a seemingly more accurate communicable signal to reach users. These signals, ranging across 3–5 octaves of sound, are distinguishable enough for those without visual impairment, so it is also likely they will serve equally as well, if not better, for those who possess any visual impairment. Their enhanced auditory sense could lead to higher accuracy in distinguishing the 3–5-octave sounds.

The experimental results showed that the sound recognition result was 70.37%, the distance recognition result was 96%, and the indoor obstacle recognition result was 81%. The device’s functionality exhibits an awareness of distance that can be furthered in more complex scenarios. The developed assistive device will produce different sounds depending on the distance and position of the obstacle in front of it. With this device, you will be informed of the distance and location of obstacles within 200 cm in uncontrolled environments. This can add to the growing knowledge in assistive technology for the daily living activities of people with visual impairment as well as enhance their productivity in routine works.
